# Somatic mutation and clonal expansions in human tissues

**DOI:** 10.1186/s13073-019-0648-4

**Published:** 2019-05-28

**Authors:** Inigo Martincorena

**Affiliations:** Wellcome Sanger Institute, Hinxton, Cambridgeshire, CB10 1SA UK

## Abstract

Recent sequencing studies on healthy skin and esophagus have found that, as we age, these tissues become colonized by mutant clones of cells carrying driver mutations in traditional cancer genes. This comment summarizes these findings and discusses their possible implications for our understanding of cancer, ageing, and other diseases.

## Mutant cell colonization in healthy skin and esophagus

Somatic mutations accumulate inexorably in our cells as we age. Most of these mutations are harmless and accumulate passively. Yet, some somatic mutations that occur early in development can cause developmental disorders, and the accumulation of somatic mutations is responsible for cancer and might contribute to ageing. Despite its importance, our understanding of somatic mutation in normal tissues had remained obscure until recently because of technical difficulties in detecting mutations that are present in small numbers of cells.

In the 1990s, studies using p53-immunostaining reported the existence of small groups or clones of a few hundred cells carrying mutations in *TP53* in sun-exposed skin [1]. Guided by these clone sizes, in 2015, we used deep targeted sequencing of small biopsies of epidermis to comprehensively detect mutant clones in normal skin from four donors aged 55–73 years [2]. This study revealed many surprises. On average, each cell in sun-exposed skin carried over 10,000 somatic mutations, most of which displayed the distinct signature of ultraviolet mutagenesis. More unexpectedly, we found evidence of positive selection of somatic mutations in at least six cancer genes, revealing that when mutations occur in key cancer genes, proliferation of these cells accelerates, leading to clonal expansions. We found that by middle age, sun-exposed skin is made up of thousands of such clones with one in every four cells carrying a positively selected mutation in a cancer gene.

**Fig. 1:**
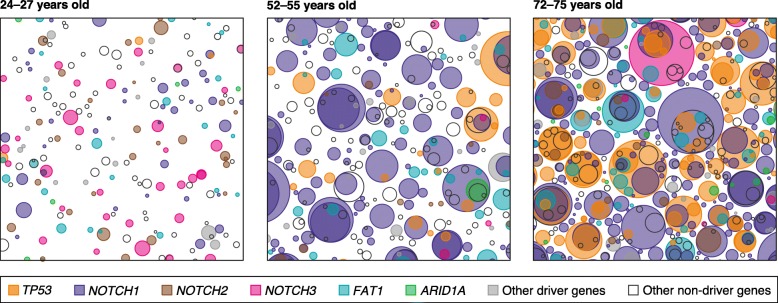
Mutant cell colonization of healthy esophageal epithelium with age. Each panel is a schematic illustration of a representative 1 cm^2^ area of normal esophagus from three donors. The younger donor was a moderate smoker and the two older donors were non-smokers. Mutant clones are shown as circles randomly distributed in space. The number of mutant clones and their sizes are directly inferred from the sequencing data, with clone areas being estimated from the fraction of sequencing reads carrying each mutation in each sample (adapted from [3])

Although these findings were striking, it seemed likely that sun-exposed skin was an exceptional tissue due to a lifetime of damage by ultraviolet light. To explore this, we then applied an analogous approach to study the mutational landscape in healthy esophagus in individuals ranging from 20 to 75 years of age [3]. To our surprise, we found that although the mutation rate in esophageal epithelium was ten-fold lower than that in sun-exposed skin, consistent with the absence of ultraviolet exposure, positive selection was stronger, leading to clones carrying mutations in cancer genes colonizing the majority of the esophagus by middle age. We detected positive selection driving clonal expansions in at least 14 cancer genes, with a density of several hundred densely packed mutant clones per square centimeter by middle age (Fig. 1). Remarkably, in most of the middle-aged and elderly patients studied, mutations in *NOTCH1* and *TP53* were found in more than 30% and in 5–20% of the cells, respectively. In an independent study published some weeks later, Yokoyama et al. [4] used exome and genome sequencing of histologically normal esophageal epithelium and reported very similar findings in a larger cohort of 139 healthy donors and cancer patients. The accumulation of somatic mutations was found to be most strongly associated with age [3, 4], with additional effects of heavy smoking and alcohol consumption [4]. This is consistent with the epidemiology of esophageal squamous carcinomas and it offers an early example of how sequencing studies of normal tissues can yield mechanistic insights into the mode of action of epidemiological risk factors.

These studies, together with the discovery of clonal hematopoiesis in blood [5] and recent reports and preprints on other tissues, including endometrium and colon [6–10], are rapidly changing our understanding of somatic mutation in normal human tissues.

## Clonal expansions and models of carcinogenesis

The high frequency of cancer-driving mutations in normal tissues may lead some to believe that somatic mutation and clonal selection are not sufficient to explain cancer development and that other factors must be required to initiate cancer. Strictly speaking, however, these results are still consistent with the traditional multi-stage model of carcinogenesis. Although we see large numbers of mutations in cancer genes in normal skin and esophagus, these mutations occur in different clones scattered throughout the tissue. Most cells carry no or one driver mutation and the frequency of driver mutations is such that the probability of a single cell carrying the right combinations of multiple driver events does not appear incompatible with the incidence of cancer in the general population. This is, in part, due to the small size of mutant clones, an important aspect that has attracted relatively little attention. Clonal expansions increase the size of the target population of each subsequent driver mutation, so under a simple model of mutation accumulation, the probability of a clone acquiring multiple driver mutations increases rapidly (geometrically) as a function of clone sizes. Better understanding of the factors that limit clonal growth in normal tissues may provide valuable insights into protective mechanisms against cancer. Further, normal cells in the tissues studied to date show considerably lower mutation rates and a more limited repertoire of mutational signatures than the corresponding cancer cells. A particularly striking difference between normal cells and cancer cells seems to be the extent of aneuploidy and chromosomal instability. Normal cells in most tissues studied to date are largely diploid, with only occasional copy-number changes [2–4, 6, 9, 10], whereas the corresponding cancers typically show extensive aneuploidy. This suggests that structural driver mutations may be an important rate-limiting step in the evolution of many tumors. Notwithstanding the importance of other factors in cancer evolution, the observation of large numbers of cancer-driving mutations in normal tissues is not inconsistent with the traditional model of cancer development by progressive acquisition of driver mutations.

Perhaps the most unexpected finding in Martincorena et al. [3] and Yokoyama et al. [4] was the observation that, by middle age, more than 30% of the cells in normal esophagus carry mutations inactivating the *NOTCH1* gene. *NOTCH1* had been assumed to be a canonical driver of esophageal squamous carcinoma as it is mutated in around 10% of cancers. Other explanations are possible, but it is conceivable and even likely that the mutation of some genes favors clonal expansions within normal tissues without increasing (or even decreasing) the risk of further evolution into cancer. Somatic evolution is not a linear road towards cancer and some mutations that drive clonal expansions may blindly push cells down evolutionary paths away from cancer. Importantly, this suggests that clones with different tumorigenic potentials compete for space within ageing tissues. Interventions that favor the growth of more benign clones at the expense of those at higher risk of transformation might thus be beneficial [3].

## Impact of somatic mutations beyond cancer

Beyond their implications for cancer, these studies raise questions about the role of somatic mutations and clonal expansions in ageing and in different diseases. Somatic mutations have long been considered a possible factor contributing to ageing. However, as with other forms of molecular damage postulated to contribute to ageing, such as epigenetic drift, telomere shortening, or protein aggregation, mutations have largely been assumed to be deleterious to the carrying cell, progressively reducing cellular fitness. The discovery of widespread clonal expansions taking over human tissues offers a powerful alternative mechanism. Positive selection at the cellular level favors changes that are beneficial to individual cells, independently of their impact on the organism. The accelerated spread of mutant clones across tissues with age may be expected to progressively compromise tissue function, contributing to ageing and to the pathogenesis of other diseases not currently linked to somatic mutations.

## Conclusions and future directions

These studies have unveiled a hidden and unexpected world of somatic evolution and clonal competition in our tissues as we age. Although striking, the high frequency of mutant clones carrying one or two cancer-driver mutations in healthy tissues is not inconsistent with the traditional multi-stage model of carcinogenesis. However, certain observations, such as the higher frequency of *NOTCH1* mutations in normal esophageal cells than in esophageal cancers, challenge commonly held assumptions and may call for some revision of our current lists of cancer-driving genes. The widespread presence of cancer-driver mutations in healthy tissues also highlights the challenges faced by early cancer detection based on liquid biopsies, but also suggests promising avenues, such as focusing on more discriminatory driver genes, combinations of mutations, and copy-number changes. Another intriguing translational opportunity raised by these studies is the possibility of interventions harnessing clonal competition to eliminate particularly tumorigenic clones from healthy tissues, an area that deserves further investigation.

We have caught a fascinating first glimpse of the extent of somatic mutation in human tissues, but much remains to be done to characterize this phenomenon fully, across tissues, across individuals, across different conditions, and across species. After decades in the dark, the next few years promise to transform our understanding of somatic mutation and clonal selection and to unravel their impact on ageing and disease.
